# Misalignment-Resilient Propagation Model for Underwater Optical Wireless Links †

**DOI:** 10.3390/s23010359

**Published:** 2022-12-29

**Authors:** João H. Araújo, Joana S. Tavares, Veridiano M. Marques, Henrique M. Salgado, Luís M. Pessoa

**Affiliations:** 1 INESC TEC—Institute for Systems and Computer Engineering, Technology and Science, 4200-465 Porto, Portugal; 2 Faculty of Engineering, University of Porto, 4200-465 Porto, Portugal

**Keywords:** underwater, optical, wireless, propagation, misalignment tolerance, Monte Carlo simulation, multiple-lens receiver, random forest

## Abstract

This paper proposes a multiple-lens receiver scheme to increase the misalignment tolerance of an underwater optical wireless communications link between an autonomous underwater vehicle (AUV) and a sensor plane. An accurate model of photon propagation based on the Monte Carlo simulation is presented which accounts for the lens(es) photon refraction at the sensor interface and angular misalignment between the emitter and receiver. The results show that the ideal divergence of the beam of the emitter is around 15° for a 1 m transmission length, increasing to 22° for a shorter distance of 0.5 m but being independent of the water turbidity. In addition, it is concluded that a seven-lense scheme is approximately three times more tolerant to offset than a single lens. A random forest machine learning algorithm is also assessed for its suitability to estimate the offset and angle of the AUV in relation to the fixed sensor, based on the power distribution of each lens, in real time. The algorithm is able to estimate the offset and angular misalignment with a mean square error of 5 mm (6 mm) and 0.157 rad (0.174 rad) for a distance between the transmitter and receiver of 1 m and 0.5 m, respectively.

## 1. Introduction

Exploration of the sea, either for monitoring and data collection or for industrial exploitation of sea resources, including sea mining, is an important area of application with a potentially strong impact on society, in line with the United Nations Sustainable Development Goals [1]. This interest is essentially motivated by the fact that existing natural resources are becoming increasingly scarce, with it being of paramount importance to guarantee access to and use of marine resources. This is especially relevant to countries with large coastal areas, such as Portugal, which is the 5th European country and 20th in the world with the largest exclusive economic zone, detaining a maritime area of more than 1.7 million km^2^ [2].

**Figure 1:**
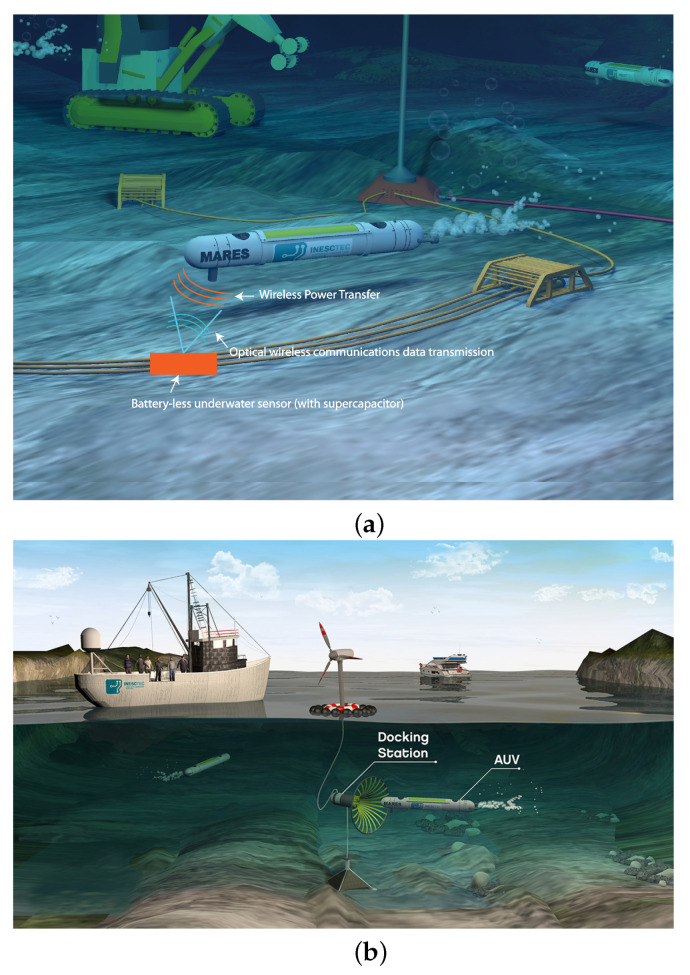
(**a**) Underwater wireless energy and communications enabling a long-term deep-sea presence. (**b**) Underwater wireless data transfer from the AUV to the docking station.

To effectively achieve this purpose, undersea communications are of paramount importance for different applications, such as the transference of data from the underwater sensors integrated into underwater wireless sensor networks (UWSNs) to autonomous underwater vehicles (AUVs), as represented in Figure 1a, or the download of large amounts of data from the AUVs to the docking stations, which are collected from the UWSN after a mission, as illustrated in Figure 1b.

In this scenario, underwater optical wireless communication (UOWC) in the blue and green region of wavelengths plays a vital role in pursuing this objective, albeit while supporting relatively short distances, allowing for the development of low-cost, low-latency, high-bandwidth, and high-robustness transmission systems, which find relevant applications [3]. Other concurrent wireless technologies for use underwater are acoustic and radio-frequency communications. The former one, despite providing long-distance transmission, suffers from low bandwidth and signal distortion due to the Doppler effect, and it is also affected by multi-path propagation that leads to inter-symbol interference. The latter is limited in bandwidth due to the scarcely available spectrum and also severely degraded by the attenuation resulting from the high conductivity and permittivity of saline water [3]. Hence, these two technologies fall short of accommodating applications that require high-speed communications.

Nevertheless, UOWC is very challenging since optical signals are affected by misalignment [4,5], ocean turbulence [6,7] and the absorption existing in the underwater channel, aside from scattering due to the presence of suspended particles, especially in turbid waters [8,9]. Even though UOWC is the most suitable model for the scenarios depicted in Figure 1, practical systems still need to be improved with increasingly innovative tools and techniques. For example, the UOWC system must be misalignment-tolerant (i.e., a sub-system capable of adjusting the emitter’s divergence (to one which the system is most tolerant to the offset) is required to maintain a stable and reliable communication link).

In this light, it is imperative to design sophisticated models increasingly capable of simulating a real underwater system. The development of these models is of extreme importance, as they can provide comprehensive knowledge of underwater channel operation, namely photon propagation in different water types, the effect of misalignment caused by water turbulence or the difficulty of maintaining the position of the transmitter or receiver, allowing one to obtain relevant guidelines such as the ideal link geometry and divergence of the emitter, contributing actively with valuable insights to the practical implementation of these systems.

To simulate the underwater channel, simple models generally do not capture the full underlying phenomena, such as the Beer–Lambert law, and hence they are not accurate, as they may not account for the real detected power. For example, this model assumes absolute alignment between the transmitter and receiver (which clearly does not match the scenario depicted in Figure 1, as it is challenging to maintain the AUV’s position due to the presence of currents and tides) and also does not account for the photons that undergo scattering but are still detected by the receiver (which greatly underestimates the detected power, especially in turbid waters). Contrarily, an accurate model for simulating underwater environments is the radiative transfer equation (RTE) [10]. Yet, as this involves nonlinear integro-differential equations (such as the scattering phase function), it is very difficult to solve analytically, and thus numerical procedures such as Monte Carlo simulations are preferred.

In our previous work [11,12], the RTE was numerically solved through a Monte Carlo simulation which accurately predicted the detected power and performed an estimation of the channel bandwidth for four water types and different beam divergences. In this work, our primary focus is to design a model that can account and compensate for a misalignment between the transmitter and receiver. To increase the misalignment tolerance, we simulated a multi-sensor-lens pair scheme in a model based on a Monte Carlo simulation of photon propagation underwater. This system, which additionally models the photon refraction at the lenses’ interface, allowed us to obtain novel results for angular misalignments and different sensor configurations based on the circle packing in a circle algorithm.

It is pertinent to understand how lateral or angular offsets will impact the signal strength, which needs to be foreseen in an actual system design. The maximum deviation in the Rx or Tx position to avoid misalignment can be assessed by calculation of the maximum allowed lateral offset (MALO). The MALO of the emitter or sensor has been previously analyzed in the literature [4,5,13,14], as the maximum offset complies with a minimum required received optical power. Here, we obtain the MALO even when the transmitter or receiver may be angularly misaligned. These works considered emitter beams with divergence, treating the sensor as a simple aperture. However, to the best of the authors’ knowledge, Monte Carlo-based simulation for accurate treatment of lens behavior does not appear in the literature. Moreover, we consider each sensor as a lens and sensor pair located at the lens focal distance from each other to improve the light collected by the sensor. We present several sensor architectures with a variable number of sensor-lens pairs and evaluate the performance in terms of position and rotation misalignment of the sensor with the emitter beam. Moreover, based on this multiple-sensor configuration and specifically the light power distribution at the receiver, defined by the power at each lens-sensor pair, we propose a random forest machine learning method to predict the lateral offset and angle of the AUV in relation to a fixed sensor configuration.

The remainder of this paper is organized as follows. Section 2 presents the implemented Monte Carlo simulation model, with a thorough description of the features added and the photon propagation conditions leading to the ulterior validation of the algorithm based on experimental results from the literature. Moreover, several sensor architectures are studied, and proper treatment of the lens behavior is developed, leading to identification of the optimal sensor scheme, and the parameters considered in the simulations are shown. The simulation results for different sensor schemes, water types, propagation distances, and angular misalignment, which are described in Section 3, where several communication scenarios are assessed, and some analysis of the system limitations that permit communications engineers to build their systems more efficiently are shown. Section 4 addresses the live misalignment offset prediction system based on the random forest machine learning technique. The final conclusions and remarks are given in Section 5.

## 2. Underwater Photon Propagation Model

The propagation of photons underwater is modeled by the RTE, which is based on the law of conservation of energy of an underwater beam of light and takes into account all losses and gains of the initial beam. Its formalism is based on the definitions of the water properties and the radiance of the beam. In its standard form, this model yields [11]

(1) $$\cos\left(\theta \right)\;\cdot \;\frac{dL\left(\theta ,\phi \right)}{cdz}=-L\left(\theta ,\phi \right)+\omega_{0}\int_{4\pi }\tilde{\beta }\left(\theta^{\prime },\phi^{\prime }\to \theta ,\phi \right)\;\cdot \;L\left(\theta^{\prime },\phi^{\prime }\right)d\Omega \left(\theta^{\prime },\phi^{\prime }\right)+S\left(\theta ,\phi \right),$$

where *c* is the beam attenuation coefficient (from the Beer–Lambert law), $\omega_{0}$ is the single scattering albedo, $\tilde{\beta }$ is the scattering phase function, *S* represents the combined path function for inelastic scattering and spontaneous emission and the radiance of the beam, *L*, is given in units of W m^−2^ sr^−1^ nm^−1^ by

(2) $$L\left(\theta ,\phi \right)=\frac{P\left(\theta ,\phi \right)}{\Delta A\;\cdot \;\Delta \Omega \;\cdot \;\Delta \lambda },$$

where *P* is the spectral radiant power, ΔA represents the cross-sectional area of the detector, ΔΩ is the solid angle in the direction of $\left(\theta ,\phi \right)$ and *z* is the distance along the beam propagation direction. Note that *L*, *c* and $\tilde{\beta }$ also depend on *z* and λ, and these dependencies are not represented here for the sake of simplicity.

The simulation of UOWC systems using the Monte Carlo algorithm is the simplest method for solving the photon propagation phenomena since, due to the complexity of the RTE equation and the problem configuration, analytical solutions only exist for very simple cases [15]. This statistical method based on the Monte Carlo algorithm mimics the physical behavior of individual photons underwater and is far more accurate than the Beer–Lambert law, which underestimates the received optical power due to assuming that scattered photons are completely lost. In this algorithm, photons are viewed as particles that move through the underwater medium with a given path length between events. These events are absorption of or collision with suspended particles in the water that scatters the photon. These are the two major inherent optical properties (IOPs) identified by Mobley for light propagation [10].

### 2.1. Monte Carlo Algorithm

In this sense, two different probability density functions provide the photon path length between events and the scattering angle after a given collision. In developing this statistical algorithm, some assumptions were made: (1) the underwater medium was homogeneous (i.e., the optical medium had a uniform composition throughout it, and the scattering and absorption effects did not have a spatial dependency); (2) only elastic scattering existed (i.e., when the scattering occurred, there were no shifts in wavelength and consequently no change in photon energy); (3) the photon generator was perfectly random; (4) no triple backscattering occurred (i.e., a scattered photon was not backscattered to its initial position); and (5) the simulation boundaries were perfectly absorbing (i.e., no photon propagated backward after passing the sensor plane). The first two assumptions were a result of the RTE equation used, which expresses the conservation of energy as the beam propagates underwater and accounts for the losses in the received beam due to scattering and absorption. The other three assumptions were associated with (1) the limitations of numerical methods, which are never truly random, and (2) the actual implementation of the numerical model itself, which sets some limits on the scattering phenomena, neglecting third-order scattering and less likely events. The assumptions were shown to be valid since it was shown previously by Cox that its results were in good agreement with the experiment [4].

There are several Monte Carlo approaches to solving the RTE, and their difference lies in what happens to the photon at each event (absorption, scattering or both) and how to quantify the absorption. Four equivalent methods capable of solving this problem are the albedo weight (AW) method, albedo rejection (AR) method, microscopic Beer–Lambert law (mBLL) method, and absorption-scattering path length rejection (ASPL) method [16].

In the first method, a weight factor given by the albedo coefficient is assigned at the interaction point to the traveling photon propagated through the free path length along the initial direction of propagation in order to propagate to the next one. The weight accounts for the loss of photons by absorption, and after the interaction, it continues propagating in the scattered direction with a reduced weight.

In the AR method, the path length of the photon is affected by the extraction of a random number uniformly distributed between 0 and 1. Thus, whether this number is lower or higher than the albedo coefficient, the photon is propagated again, or its trajectory is terminated, respectively. In this case, the weight applied to the photon trajectory is one.

In the mBLL method, the detected photons will favor the same trajectories independent of the absorption coefficient distribution, and the weight factor assigned to those trajectories is calculated through the exponential of the absorption coefficient integral along the whole trajectory.

The latter consists of extracting two random numbers uniformly distributed between 0 and 1, which allows us to obtain the scattering and absorption coefficients. With these, the individual path lengths are obtained and compared for the two situations. If the path length associated with the scattering event is less than or equal to the path length associated with the absorption event, then the photon is propagated, and if it is greater, then the photon is terminated. Note that for the AW, AR and ASPL methods, the actual trajectories chosen by the detected photons depend on the distributions of both the scattering and absorption coefficients [16].

In this work, the AW method was adopted since it exhibits superior convergence performance when compared with the AR and ASPL methods [16]. As referred to earlier, in this approach, each photon has a given amplitude starting at one, and each time it is absorbed, its amplitude is multiplied by a weight given by the water albedo coefficient. This approach was preferred for several research papers [13,17,18,19,20,21], although they are all equivalent [16].

#### 2.1.1. Coordinate System and Initial Conditions

In the algorithm geometry, a Cartesian coordinate system is considered where the receiver plane is situated on a fixed point in the *x*/*y* plane. The emitter is pointed in the direction *z*, which is referred to here as the optical axis. To start propagating photons through the medium, they must be given a position and a vector of propagation that properly simulates a laser beam. Photons start to propagate in the plane z=0, and their initial positions $\left(x_{0},y_{0},z_{0}\right)$ are determined from a sampled Gaussian distribution, which is more realistic than a flat distribution, with a standard deviation equal to the laser beam’s waist radius $w_{0}$ [22]:

(3) $$\rho \left(r\right)=\exp\left(\frac{-r^{2}}{2w_{0}}\right).$$

Given a sampled radius, the (x,y) positions are easily calculated as follows:

(4) $$\begin{array}{cc} x_{0} & =r\cos\left(\phi \right),\\ y_{0} & =r\sin\left(\phi \right). \end{array}$$

**Figure 2:**
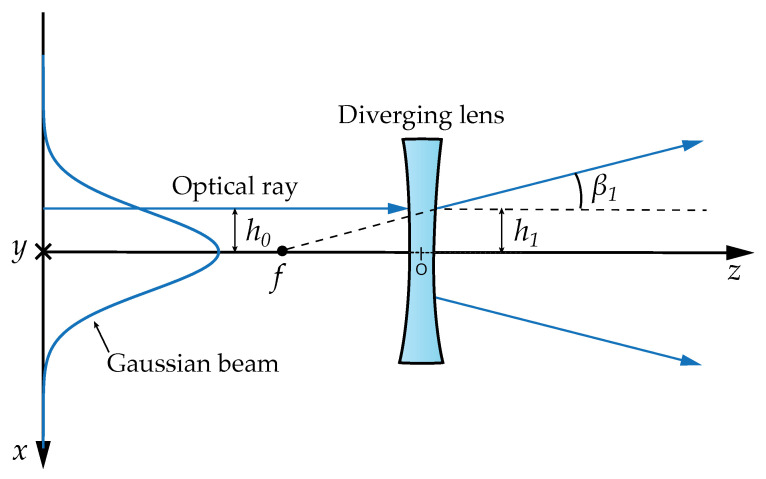
Scheme of the opening of the optical beam passing through a lens.

After obtaining the photon’s initial position, one needs to simulate the emitter’s divergence to calculate the initial direction for each photon. A hypothetical lens located at the emitter side next to the laser provides the mathematical basis for defining the beam divergence. The focal distance $f_{l}$ of this lens will depend on the desired half-angle beam divergence $\psi_{div}$ (represented in Figure 2) and on the laser beam waist radius $w_{0}$, and it is given by

(5) $$f_{l}=\frac{w_{0}}{\psi_{div}}.$$

The initial polar angle of each photon is obtained through the ray transfer matrix analysis using the paraxial approximation. The optical ray coordinates are defined as (h,β), where *h* is the height and β is the slope angle relative to the optical axis. The effect of the lens on the initial ray coordinates is obtained by multiplication with the optical element matrix of the lens, given by [4]

(6) $$\left[\begin{array}{c} h_{1}\\ \beta_{1} \end{array}\right]=\left[\begin{array}{cc} 1 & 0\\ \frac{-1}{f_{l}} & 1 \end{array}\right]\left[\begin{array}{c} h_{0}\\ \beta_{0} \end{array}\right].$$

**Figure 3:**
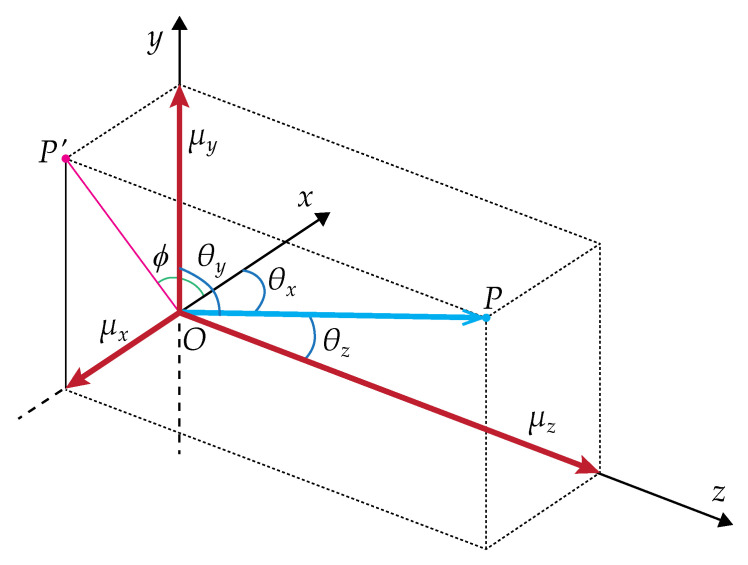
Scheme of direction cosines. The blue vector depicts the photon’s direction vector, and the red vectors ($\mu_{x}$, $\mu_{y}$, $\mu_{z}$) are the projections of the photon’s direction vector onto the *x*, *y*, and *z* axes.

For each photon, the initial azimuthal angle $\phi_{0}$ is taken randomly from [0,2π] since the beam is radially symmetric, which is accomplished by taking a random number *q* uniformly distributed between [0,1] and multiplying it by 2π. Given a certain point *P* in space with an origin *O*, the azimuthal angle is defined as the angle between the positive *x* axis and the projection of the line segment $\bar{OP}$ on the *x*/*y* plane, as illustrated in Figure 3.

The photon direction vector is divided into its projections on the three axes in order to avoid complex and time-consuming running trigonometric functions. These projections $\mu_{x}$, $\mu_{y}$ and $\mu_{z}$ are called the direction cosines, being defined by

(7) $$\begin{array}{cc} \mu_{x} & =\cos\left(\theta_{x}\right),\\ \mu_{y} & =\cos\left(\theta_{y}\right),\\ \mu_{z} & =\cos\left(\theta_{z}\right), \end{array}$$

where $\left(\theta_{x},\theta_{y},\theta_{z}\right)$ are the angles between the propagation vector and the Cartesian axes, which must comply with the normalization $\mu_{x}^{2}+\mu_{y}^{2}+\mu_{z}^{2}=1$.

#### 2.1.2. Photon Propagation

The photon path length is the distance the photons travel in the homogeneous medium between interactions, which can be absorption, scattering, or both. The probability of a photon traveling over an optical scattering path of a length *l* is given by [10]

(8) $$P_{l}\left(l\right)=1-e^{-l}.$$

A typical method to obtain a sample from a given probability distribution function is the inverse transform sampling [23]. Since Equation (8) is an invertible function, to obtain a sample, one simply needs to invert it:

(9) $$l=-\ln\left(1-P_{l}\left(l\right)\right).$$

Then, we add a random, uniformly distributed number between [0,1], represented here as $P_{l}\left(l\right)$. Hence, the optical path length is then defined as

(10) l=c·r,

where *c* is the attenuation coefficient and *r* is the geometric distance between optical events.

Finally, it comes naturally that *r* yields

(11) $$r=-\frac{1}{c}\ln\left(1-P_{l}\left(l\right)\right).$$

Regarding the scattering, each successive scattering event causes the trajectory to rotate its local coordinate frame by $\left(\theta ,\phi \right)$. Given a volume scattering function $\tilde{\beta }$, the new angle $\theta^{\prime }$ is given by solving the following equation:

(12) $$q=\int_{0}^{\theta^{\prime }}\tilde{\beta }\left(\theta \right)\sin\left(\theta \right)d\theta ,$$

where *q* is another random, uniformly distributed number between [0,1]. The new azimuthal angle $\phi^{\prime }$ is independent of any probability density function and therefore is sampled uniformly between [0,2π].

#### 2.1.3. Validation of the Algorithm

The photon propagation algorithm validity was confirmed by comparing it with experimental results for on-axis laser beam propagation through a closed 3.6 m water tank taken by Cox and Muth [4].

The communication system was composed of an emitter, a 532 nm diode-pumped solid-state laser with a 2 mm beam diameter and divergence of 1.5 mrad and a receiver composed of a 25.4 mm achromatic biconvex lens at a 75 mm focal distance of a photomultiplier tube (PMT) unit with an 8 mm active area. The laser was considered collimated in the simulation, and the different levels of water attenuation coefficients (water types) were obtained by adding Maalox as a scattering agent.

**Figure 4:**
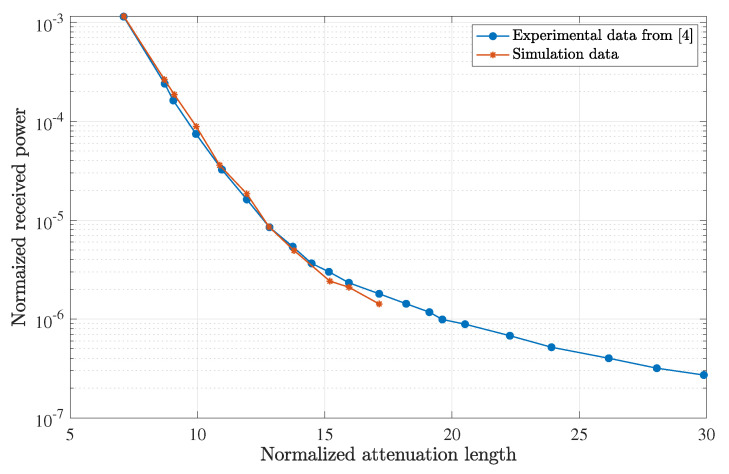
Comparison of simulation results for the normalized received power with Cox and Muth’s experimental data for a 3.6 m water tank, with different attenuation coefficients made by adding different quantities of scattering agent Maalox.

This simulated scenario is the same one in which Cox and Muth’s code was validated experimentally. The average power was measured as a function of the attenuation coefficient. The normalized intensity power was plotted versus the attenuation coefficient in Figure 4. Good agreement can be seen for all the points at which the simulation was performed.

### 2.2. Emitter and Sensor Rotation

**Figure 5:**
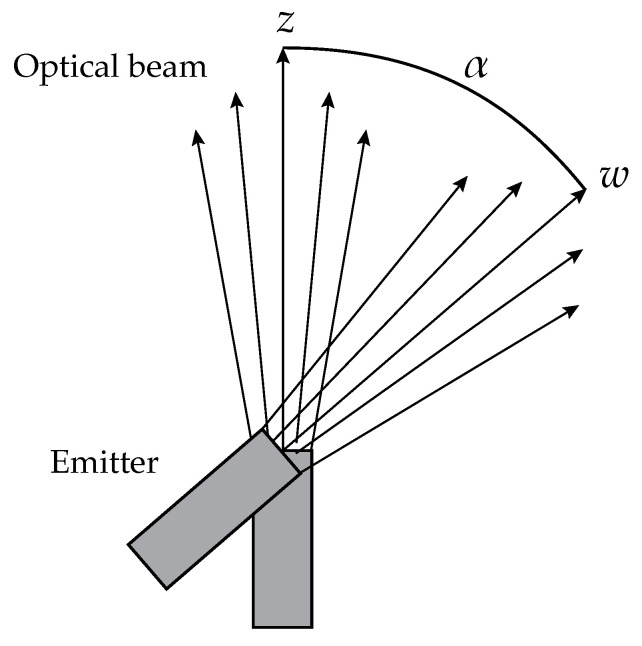
Rotation of the emitter direction from the default $\hat{z}$ direction to any desired vector w.

The emitter was frequently pointed along the *z* axis in the literature [13]. Yet, in this work, the ability to rotate the emitter along any direction when given a direction vector *w*, as represented in Figure 5, was introduced.

An efficient routine with no square roots or trigonometric functions to speed up the running time was implemented to generate a three-dimensional rotational matrix that rotated any unit vector *f* into *w* at an angle α [24]. Here, we considered *f* to be the unit vector on the *z* axis, which was defined as $\hat{z}$. The vector product of $\hat{z}$ and *w* was defined as s with vector components $\left(s_{x},s_{y},s_{z}\right)$:

(13) $$s=\hat{z}\times w,$$

In addition, the dot product of $\hat{z}$ and *w* is expressed as

(14) $$q=\hat{z}\;\cdot \;w.$$

An auxiliary variable *m* is defined as

(15) $$m=\frac{1-q}{1-q^{2}}=\frac{1-q}{s\;\cdot \;s},$$

Then, after considerable algebra, the rotation matrix can be simplified to [24]

(16) $$R\left(f,w\right)=\left[\begin{array}{ccc} q+ms_{x}^{2} & ms_{x}s_{y}-s_{z} & ms_{x}s_{z}+s_{y}\\ ms_{x}s_{y}+s_{z} & q+ms_{y}^{2} & ms_{y}s_{z}-s_{x}\\ ms_{x}s_{z}-s_{y} & ms_{y}s_{z}+s_{x} & q+ms_{z}^{2} \end{array}\right].$$

**Figure 6:**
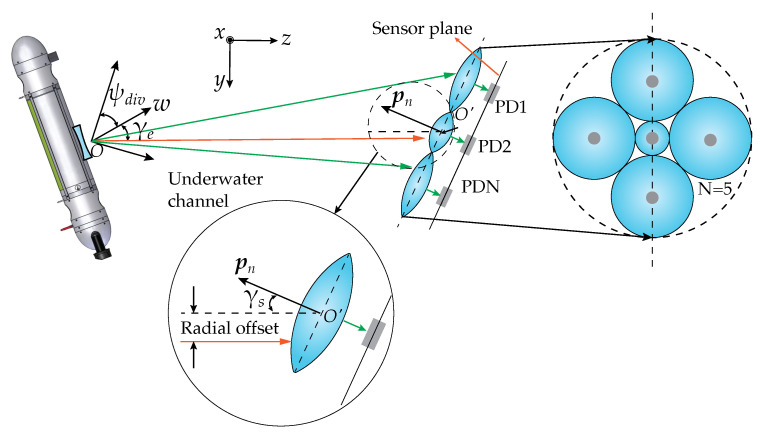
AUV communicating with fixed receiver. Both emitter and receiver are rotated in relation to the *z* axis by the angles $\gamma_{e}$ and $\gamma_{s}$, respectively.

Figure 6 shows a diagram of the complete system, including the AUV, diverging beam, propagation channel and receiver, composed of *N* lenses and detectors. The figure shows an example of angularly misaligned communication, in which the rotation angle of the emitter and the plane of the sensors are given by $\gamma_{e}$ and $\gamma_{s}$, respectively.

### 2.3. Channel Propagator

The photons were propagated using the Monte Carlo algorithm presented in Section 2.1. To be able to simulate an architecture of sensors in a plane rotated with regard to the emitter, the condition of termination of each photon had to be changed. Instead of finishing the photons in a given finished plane $z=z_{f}$, they were finished in a general plane given by its normal vector $p_{n}$$\left(p_{nx},p_{ny},p_{nz}\right)$. Thus, the photons’ new position $\left(x^{\prime },y^{\prime },z^{\prime }\right)$ was evaluated after every displacement. In the case where the photon was past the plane, the following condition

(17) $$p_{nx}x^{\prime }+p_{ny}y^{\prime }+p_{nz}z^{\prime }>cz_{f},$$

would be verified. Then, the intersection with the plane was calculated, and the photons’ data (i.e., the position in the last plane, the vector of propagation, and the weight) would be saved in a file for later analysis.

### 2.4. Sensor Architecture

In this work, different sensor architectures are studied, and all are composed of a different number of lens-sensor pairs located at different positions inside a circle. The total output signal power is obtained by adding the powers at each detector. Aside from providing additional sensitivity, this architecture gives additional information about the lateral offset between the transmitter and receiver (see Figure 6), which will be explored later in Section 4, for prediction of the misalignment.

#### 2.4.1. Accurate Lens Behavior

An innovative feature was added to the Monte Carlo algorithm: the ability to simulate accurately the refraction of photons when these pass through the lens. As the photons are treated individually in this method, one accurate method is to calculate the refraction at each surface of the lens for each photon. Mathematically, the biconvex lens used was treated as two parts of spheres with a thick section in between.

After calculating the intersection of the photons with the first interface of the sphere, the vector form of Snell’s law is applied. If one considers $\theta_{1}$ to be the angle of incidence with the normal of the sphere $g_{i}$ and $\cos\left(\theta_{1}\right)=-g_{i}\;\cdot \;v_{i}$, where $v_{i}$ is the vector of propagation of the photon, then the vector of propagation after the first refraction $v_{r1}$ is given by [25]

(18) $$v_{r1}=\frac{n_{1}}{n_{2}}v_{i}+\left(\frac{n_{1}}{n_{2}}\cos\theta_{1}-\cos\theta_{2}\right),$$

where the following definition is used:

(19) $$\sin\theta_{2}=\frac{n_{1}}{n_{2}}\sin\theta_{1}.$$

**Figure 7:**
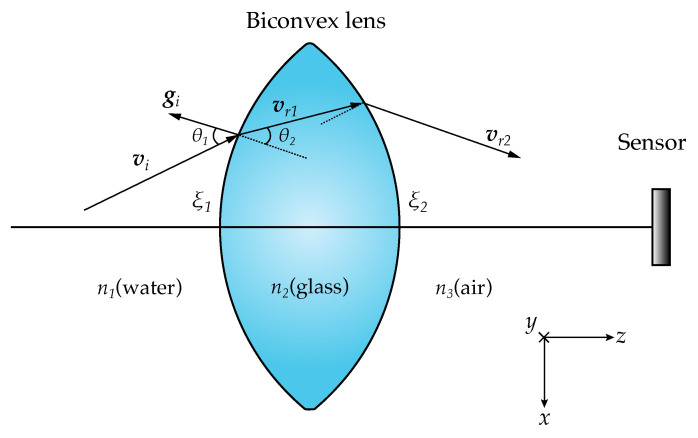
Photon as ray vector being refracted at both medium interfaces. The lens directs the photons to the sensor.

After the first refraction, on the interface defined as $\xi_{1}$ which separates $n_{1}$ from $n_{2}$, the photon is propagated through the glass until the next lens surface. Here, the photon meets another interface between $n_{2}$ and $n_{3}$, $\xi_{2}$, where the same refraction calculation is made with the new vector and refractive indexes as illustrated in Figure 7.

The Fresnel reflection or photon loss at the water–glass interface was about 0.2%, and the reflection at the glass–air interface was 3.2%, assuming normal incidence. This power loss could be compensated by increasing the transmitted power since only the ratio between the received power and the transmitted power was relevant for the calculation of the MALO and did not affect the main conclusions.

#### 2.4.2. Packing

The positions of the lenses were chosen from known results of a proven algorithm, known as “circle packing in a circle” [26], using a non-congruent approach to maximize the area occupied by the lenses. After defining a number of lenses N and finding their packing positions, another lens in the center was added with the maximum possible radius.

**Figure 8:**
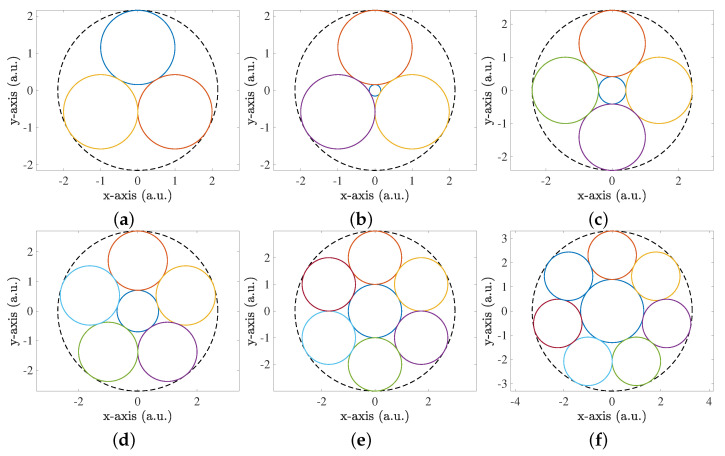
Scheme of N sensors given by the “circle packing in a circle” algorithm. (**a**) N = 3; (**b**) N = 4; (**c**) N = 5; (**d**) N = 6; (**e**) N = 7; (**f**) N = 8.

A diagram of the positions of the various lenses for a number of lenses from 3 to 8 is portrayed in Figure 8. The optimal scheme will be analyzed and discussed later in Section 3.2. A multi-lens-sensor pair configuration is discussed instead of a single lens and one sensor since it has the added advantage of providing spatial information on the transmitter and receiver misalignment, which will be explored later in Section 4.

### 2.5. Simulation Parameters

Inasmuch as this work’s purpose is to study short-range communication scenarios (such as those illustrated in Figure 1), the simulated distances between the emitter and receiver considered were 0.5 m and 1 m, respectively. The typical distances for UWOC systems used were within a range of a few tenths of meters to dozens of meters [8]. We restricted the simulation to a low range for the sake of the computing time.

Moreover, all the parameters used in the simulations are given in Table 1, and the attenuation and albedo coefficients for the different water types are presented in Table 2.

## 3. Simulation Results

In this section, we describe the simulation scenarios and present the performance assessment of each sensor architecture. The MALO is the maximum offset misalignment permitted between the transmitter and receiver that complies with a minimum ratio of received power to transmitted power. For the calculation of the MALO, the lens and sensor schemes were displaced from the central position on the *x*/*y* plane both angularly and laterally. In the following analyses, the angular misalignment is not taken into account, except in the last subsection.

### 3.1. Evaluation Algorithm

The evaluated scenarios encompassed the water type, total distance, divergence, and vector of propagation of the emitter and plane of the final sensor.

Initially, the photons were propagated using the algorithm described in Section 2.3, and the final parameters were stored in a data file, as mentioned previously. For each scenario, the received power was obtained after the receiver architecture was simulated. The algorithm is general enough to consider the case where both the transmitter and receiver are misaligned at a certain angle and simultaneously are offset between each other.

Then, we computed the received power at the sensors in each of the positions of the lenses’ scheme. Even though the receiver plane was tilted due to angular misalignment, the lens positions were shifted in the *x*/*y* plane, which made this analysis pertinent.

Afterward, another simple routine calculated the maximum radius the AUV could move for the receiver to have a minimal amount of relative received power. The minimum accepted ratio of received power to transmitted power was 1 × 10^−3^, which is equivalent to considering an emitter with a typical power of 0 dBm and a minimum received optical power of −30 dBm (suitable to achieve a forward error correction (FEC) limited bit error rate (BER) of 3.8 × 10^−3^ for a data rate of 100 Mbit/s) [27].

**Figure 9:**
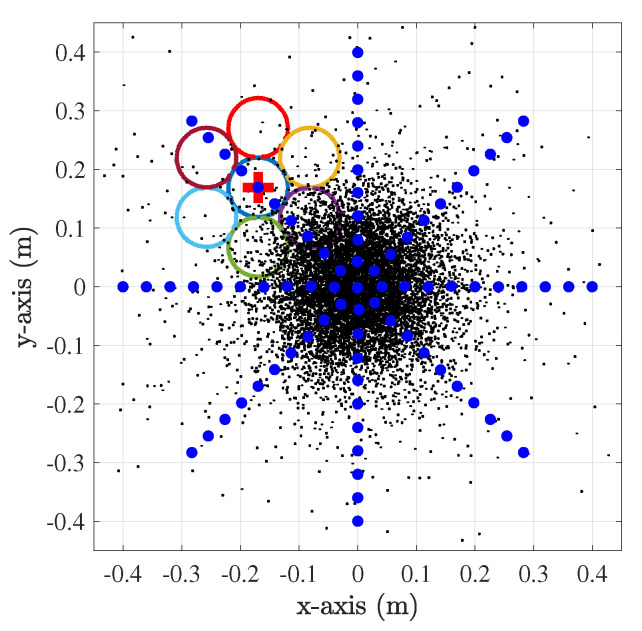
Moving scheme of sensors for MALO calculation. The sensor scheme was offset through the 2D plane radially from 0 m to 0.4 m and at angles from 0° to 360° in steps of 45°.

Figure 9 shows the scheme with seven lenses, where the blue dots mark the various positions in which the set of lenses was displaced for the calculation of the MALO. The lenses’ scheme is centered in all the blue dots’ positions for calculating the received power.

### 3.2. Sensor Scheme

Initially, the sensor architecture was analyzed, with the sensors’ positions given by a “circles inside a circle” packing algorithm, as a means to increase the tolerance to the position and angular misalignment problem. The analysis of the number of sensors of this scheme was performed for clear waters and a propagation distance of 1 m.

**Figure 10:**
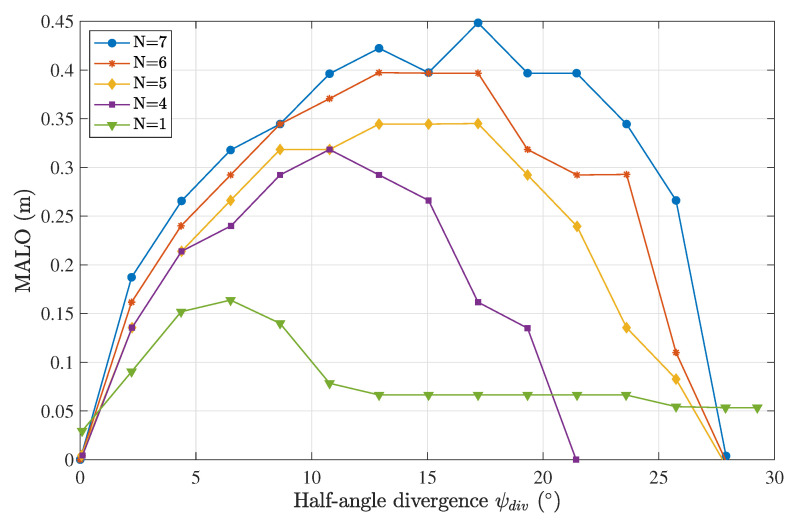
MALO calculation as a function of the divergence of the emitter for a different number of sensors in the sensor scheme, considering a clear water type and a Tx-Rx distance of 1 m.

The results shown in Figure 10 reveal a distinct improvement in the tolerance to misalignment for a higher number of sensors, mainly due to the higher total receiver area.

The seven sensors in a packing disposition, as depicted in Figure 8e, increased the MALO by approximately a factor of three compared with the one-lens-only case. In addition, the seven-sensor case was trivially optimal and conveniently required seven equally sized lenses, and thus the case corresponding to eight lenses was not simulated.

### 3.3. Water Types

The next analysis consisted of the calculation of the MALO parameter for different water types using the parameters from Table 1. The distance between the emitter and receiver was 1 m, and a seven-lens scheme was used.

**Figure 11:**
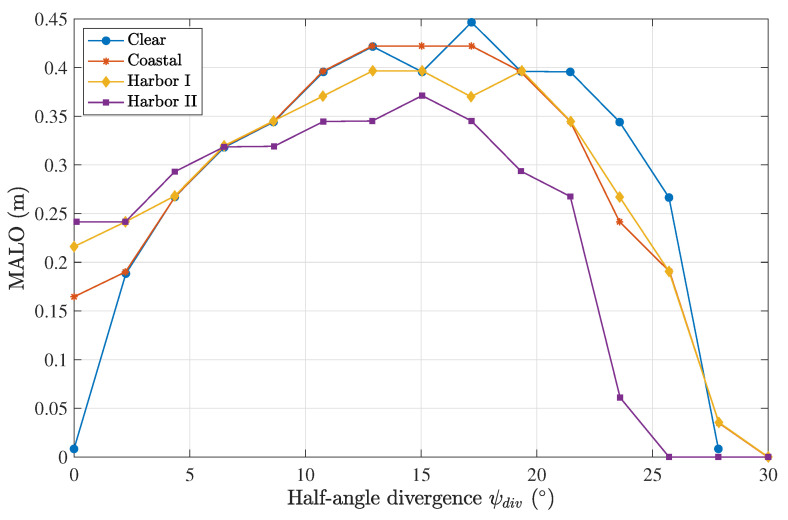
MALO calculation as a function of the divergence of the emitter for different water types, considering a seven-lens scheme and a Tx–Rx distance of 1 m.

The results are presented in Figure 11, showing a characteristic curve for each of them. These curves show the optimal divergence that maximized the tolerance to the position misalignment of the system. While this work is a comprehensive analysis of the sensor-lens pair, these results confirm the conclusions of a previously published work [14]. An evident difference between different water types was that the MALO was non-zero at a beam divergence of zero for more turbid waters and increased with the water turbidity. This outcome was expected due to increased scattering, which acted as a diverging factor, maximizing the MALO.

### 3.4. Propagation Distance

The MALO calculation was performed while sweeping the divergence using the parameters from Table 1 and a seven-lens scheme for different lengths between the emitter and receiver and for the two extremes of water turbidity: clear and harbor II waters.

**Figure 12:**
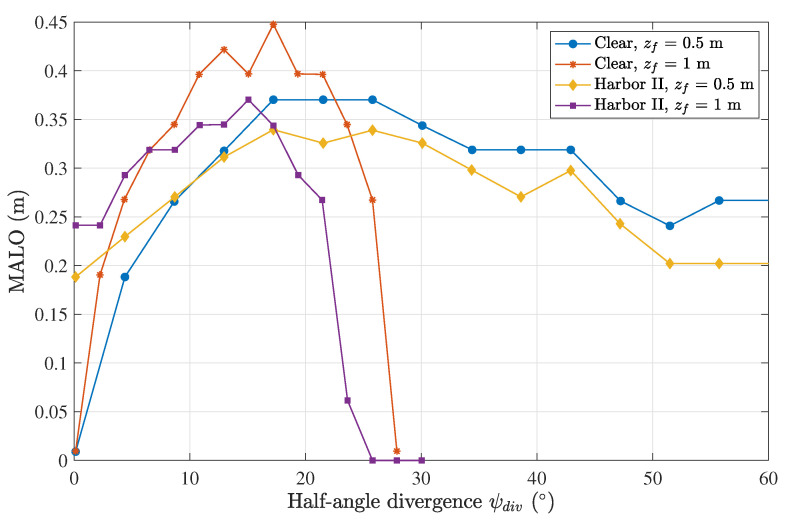
MALO calculation for different water types at different Tx–Rx distances and for varying divergence angles, considering a seven-lens scheme.

The obtained results are plotted in Figure 12, showing similar curves that exhibit an optimum divergence that depends on the distance. For a distance of 1 m, there was a larger tolerance to misalignment (higher MALO) than for 0.5 m. This can be attributed to the higher number of scattering events and the opening of the beam due to propagation. The consequence was that the maximum was obtained at a lower value of divergence and required a more accurate configuration (lower range of divergence angles) due to the rapid decay of the MALO.

On the other hand, above the ideal divergence, the MALO degraded rapidly at 1 m. Here, the increased scattering and propagation distance made the beam spread so much that the minimum amount of power did not reach the sensors. The same behavior was observed for both the clear and harbor II waters, from which we concluded that the geometry of the link and the distance, in particular, was the limiting performance factor, which could be somewhat overcome by adjusting the divergence angle. For instance, in clear waters and at a distance of 0.5 m, the MALO was 0.37 m, whereas when the distance was increased to 1 m, the MALO was actually increased to 0.45 m by adjusting the divergence angle from 22° to 17°. If the divergence was maintained at 22°, then the MALO actually decreased to 0.4 m.

### 3.5. Angular Misalignment

Lastly, the angular alignment capacity of the emitter-receiver system in highly turbid harbor II waters was assessed using a propagation distance of 0.5 m and a seven-lens scheme configuration. Three different configurations were simulated: (1) an aligned system, (2) only the emitter rotated in a given direction, and (3) only the emitter rotated in the same direction.

**Figure 13:**
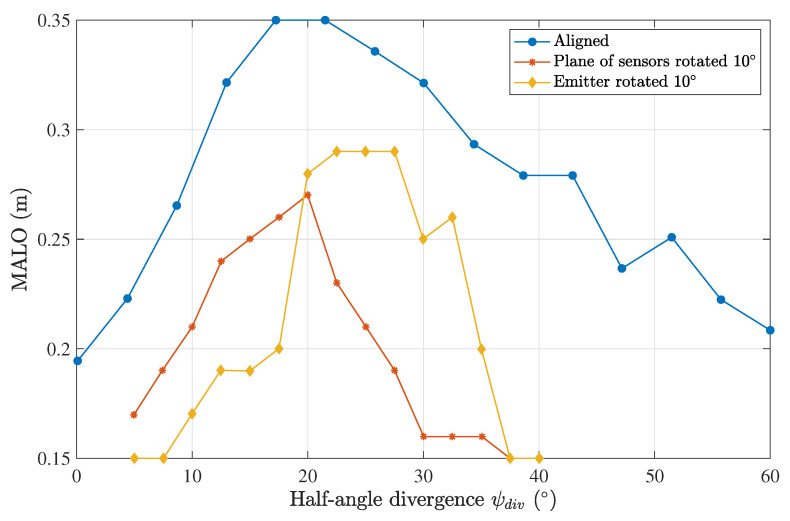
The MALO as a function of the divergence of the emitter for rotated communication links, considering a harbor II water type, a Tx–Rx distance of 0.5 m and a 7-lens scheme.

**Figure 14:**
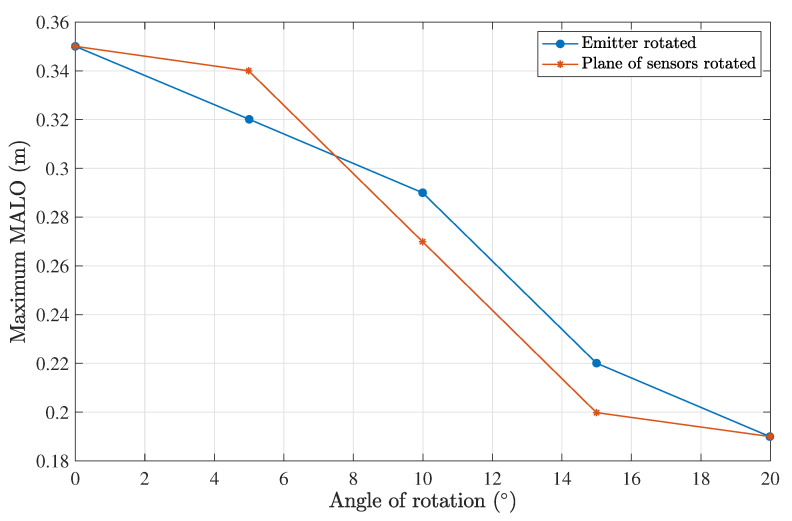
The maximum MALO as a function of the rotation angle, considering a harbor II water type, a Tx–Rx distance of 0.5 m and a 7-lens scheme.

**Figure 15:**
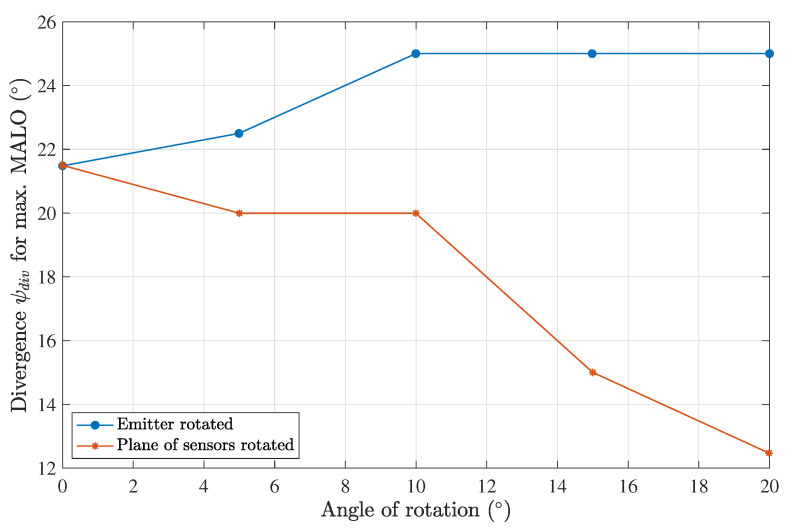
Divergence for the maximum MALO as a function of the rotation angle, considering a harbor II water type, a Tx–Rx distance of 0.5 m and a 7-lens scheme.

Figure 13 shows the results for a rotation of 10°. This analysis was performed for angles of 0°, 5°, 10°, 15°, and 20°. As expected, we observed a worse performance when either the emitter or the plane of the sensors was rotated, as shown in Figure 14. When the emitter was rotated to compensate for fewer photons arriving at the sensors, an increase in the optimal divergence was seen. The opposite behavior was observed when the plane of sensors was rotated. The optimal divergence as a function of the angular misalignment is plotted in Figure 15 for both cases.

## 4. Live Misalignment Offset Prediction

**Figure 16:**
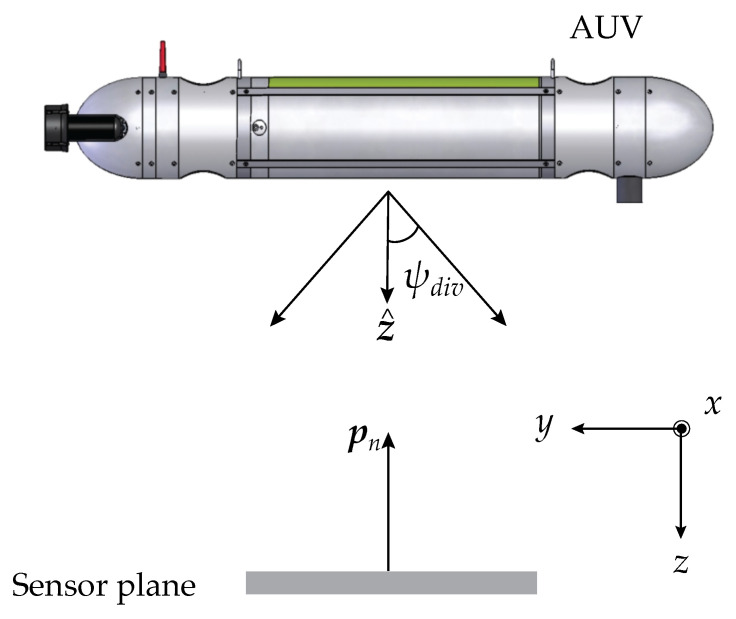
Diagram of AUV communicating with a fixed receiver using a divergent optical beam.

As seen in Section 3, in a real communication scenario between an AUV and a fixed sensor, as illustrated in Figure 16, an optimum divergence of the emitter exists at which the system is most tolerant to the offset misalignment, and the MALO takes its maximum value. To maintain a stable and reliable communication link in a live communication scenario in the presence of ocean currents, the AUV needs to adapt to the divergence of the emitter by changing the distance of the laser to the diverging lens, based on how much the present average offset is. However, the transmitter does not have this information unless it is estimated by the receiver and sent to the transmitter. Assuming that a low data rate link is established in the reverse direction, one can hypothesize that the distribution of power over the arrangement of lenses at the receiver, namely the use of seven lenses, might provide sufficient information to extract the critical misalignment data.

Based on this supposition, a random forest machine learning method was assessed for its suitability to identify the offset of the AUV in relation to the fixed sensor at each moment. With this aim, using a seven-lens scheme, a random forest algorithm was trained based on a 64 × 7 matrix containing the power received by each lens at each point of a 8 × 8-point grid in the AUV receiver plane (r,θ), which constituted the training labels for a specific transmission length and divergence angle and are presented in Table 3.

The received power at each lens was obtained from the numerical simulation by propagating $10^{9}$ photons. Having a trained algorithm with optimized parameters, its ability to estimate the offset was then tested on a different matrix holding the power received at each lens but at another set of random points in space, given in Table 4.

The optimal random forest parameters were found to be the following:

- N estimators: 400;
- Minimum samples per split: 2;
- Minimum samples per leaf: 1;
- Maximum features: square root;
- Maximum tree depth: none;
- Bootstrap: false.

**Figure 17:**
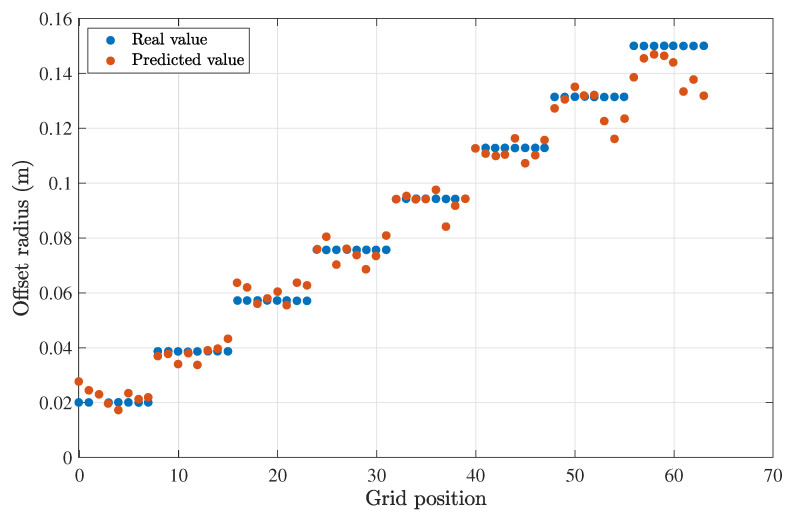
Comparison between the predicted and expected offset radius for a Tx–Rx distance of 1 m.

**Figure 18:**
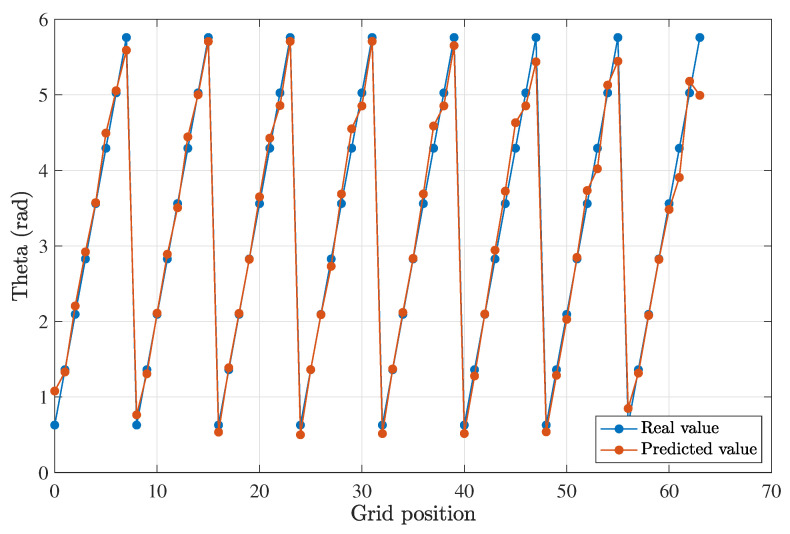
Comparison between the predicted and expected offset angle for a Tx–Rx distance of 1 m.

For a propagation distance of 1 m and a clear water type, the half-angle divergence was set to 8.5°. A comparison between the estimated results and the actual test data can be seen in Figure 17 and Figure 18. The deviation or error from the expected values was assessed by calculating the root mean squared (RMS) error of the two sets of values (predicted and estimated). The RMS error for the estimation of the radius was 6 mm, and it was 0.174 rad (10°) for the angular offset.

For a propagation distance of 0.5 m and clear waters, the optimum half-angle divergence obtained from the simulations was 10°. The RMS value for the estimation of the offset (r,θ), which in this case was 5 mm, was 0.157 rad (9°).

The results showed good agreement, confirming the initial hypothesis and that machine learning methods may be used to estimate the offset successfully. When comparing these results with the MALO calculations in Figure 10, we may conclude that an error of 5–6 mm on the lateral offset was sufficiently accurate to provide relevant information to the transmitter so that an optimum divergence was chosen, hence leading to better performance for the optical link.

## 5. Conclusions

This paper presented a simple yet powerful tool based on the Monte Carlo algorithm for modeling the propagation of photons in the underwater medium. Several novel contributions to the area of underwater optical wireless communications were reported, and new features were added to the already existing simulation algorithms. An accurate model of the beam divergence was described, supported by the analysis of lateral offset and angular misalignment between the transmitter and receiver. A receiver configuration with multiple sensors and lenses laid out on a “circle packing in a circle”, including a precise treatment of photon refraction at the lens interface, was proposed, which showed increased tolerance to misalignment. The assessment of system performance was conducted based on the maximum lateral offset for different sensor schemes, water types, propagation distances, and angular misalignments, which proved the existence of an optimal divergence in agreement with previous results.

Some relevant results were achieved: (1) the ideal divergence of the emitter’s beam was shown to be around 15°, independent of the water turbidity for a 1 m Tx–Rx distance and considering a 7-lense scheme; (2) the ideal divergence for clear waters was found to be around 17° for a distance between the Tx and Rx of 1 m, while for a length of 0.5 m, the optimum divergence was 21°; (3) the architecture of the receiver with multiple sensors arranged in a compact geometry was proven to exhibit an increase in the MALO by approximately a factor of 3; and (4) the analysis of the Tx–Rx rotation revealed that rotating the emitter increased the optimal divergence, whereas turning the plane of the sensors decreased it. Moreover, a supervised learning algorithm successfully predicted the lateral offset and angle of the AUV based on the received light power distribution of each lens in a fixed sensor configuration. There was good agreement between the results predicted by the algorithm and the simulation, with a maximum RMS error of 6 mm and 10° for the predicted values of the lateral and angle offset parameters, respectively.

The assessment of the limitations of the optical wireless underwater communication link under misalignment conditions in application scenarios involving AUVs and the identification of optimum operating conditions might be of interest to the research community and design engineers, particularly those looking to implement wireless sensor networks and the transfer of data between nodes and autonomous underwater vehicles.

Future work will include obtaining simulation results for longer distances between the transmitter and receiver and the corresponding MALO results, allowing one to assess the performance of medium-range links as well as the feasibility of the offset prediction algorithm in a real AUV scenario application.

## Figures and Tables

**Table 1:** Simulation parameters.

Parameters	Value
Number of photons	1 × 10^6^
Wavelength (λ)	520 nm
Refractive index of water ($n_{1}$)	1.33
Refractive index of lens ($n_{2}$)	1.46
Emitter (Tx) beam waist radius	1 mm
Emitter (Tx) divergence	from 0° to 60°
Sensor (Rx) radius	10 mm
Lens thickness	15.6 mm
Lens curvature radius	52.6 mm
Lens diameter	101.6 mm
Lens focal distance	60 mm
Number of lenses (*N*)	1; from 4 to 7
Distance between Tx and Rx ($z_{f}$)	from 0.5 m to 1 m
Misalignment angle between Tx and Rx	from 0° to 20°

**Table 2:** Attenuation coefficient and albedo considered for different water types [10].

Water Type	c (λ)	$\omega_{0}$
Clear waters	0.15 m^−1^	0.25
Coastal waters	0.10 m^−1^	0.55
Harbor I waters	1.10 m^−1^	0.83
Harbor II waters	2.19 m^−1^	0.83

**Table 3:** Grid of 8 × 8 points in space used for training.

*r* (cm)	1.0000	3.1428	5.2857	7.4286	9.5714	11.7143	13.8571	16.0000
θ (rad)	0.6283	1.3614	2.0944	2.8274	3.5605	4.2935	5.0265	5.7596

**Table 4:** Grid of 8 × 8 points in space used for testing the algorithm, estimating the offset between the transmitter and receiver.

*r* (cm)	2.0000	3.8571	5.7143	7.5714	9.4286	11.2857	13.1428	15.0000
θ (rad)	0.6283	1.3614	2.0944	2.8274	3.5605	4.2935	5.0265	5.7596

## Data Availability

Not applicable.
